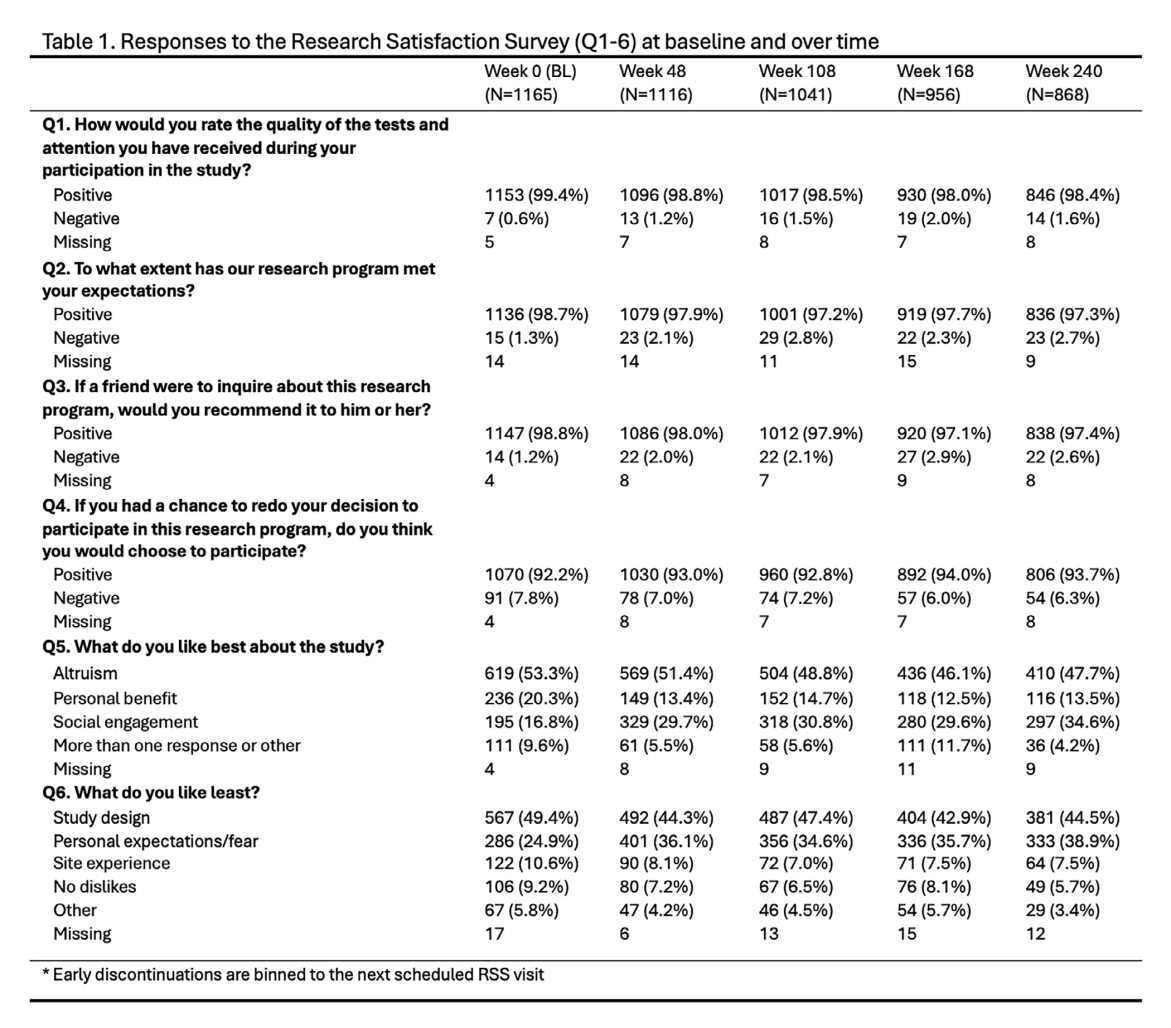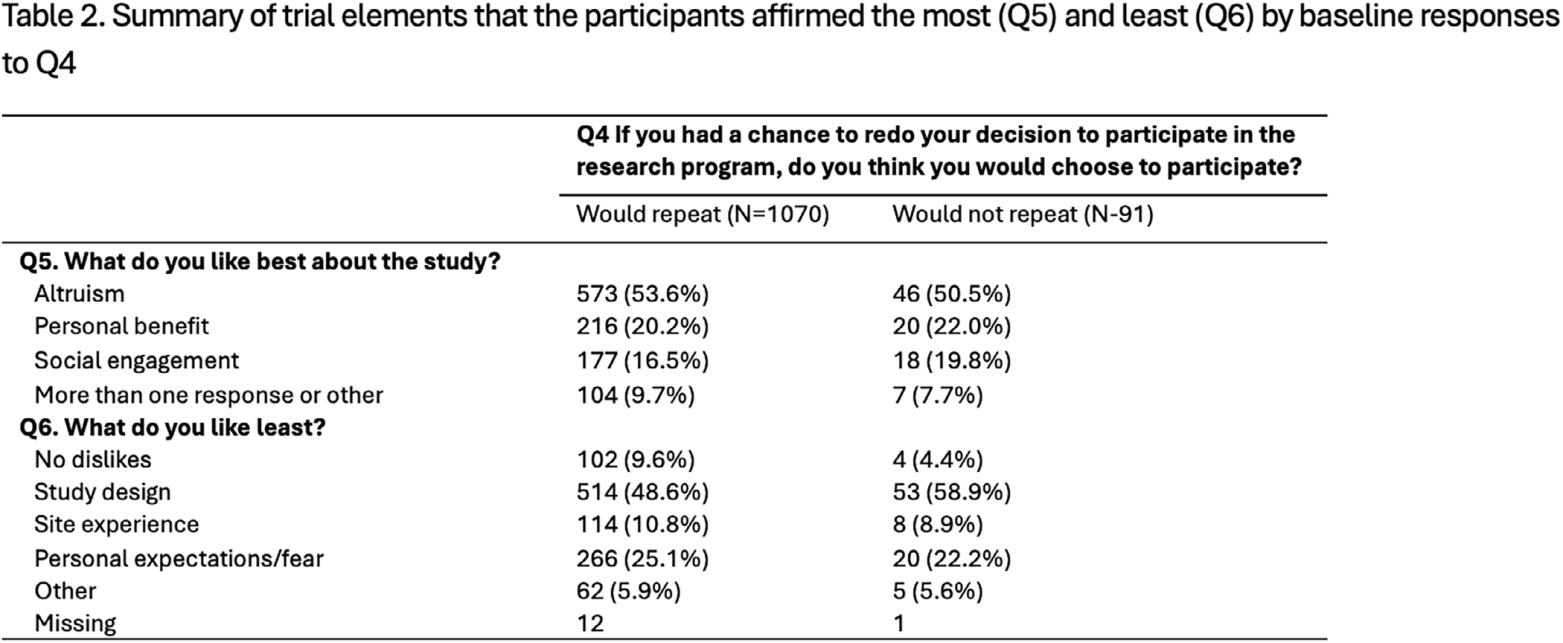# Participant satisfaction in a preclinical Alzheimer's disease trial

**DOI:** 10.1002/alz70859_103526

**Published:** 2025-12-25

**Authors:** Marina Ritchie, Kedir Hussen, Paul S. Aisen, Josh D Grill, Reisa A. Sperling, Rema Raman

**Affiliations:** ^1^ Alzheimer's Therapeutic Research Institute, University of Southern California, San Diego, CA USA; ^2^ The UC Irvine Institute for Memory Impairments and Neurological Disorders, Irvine, CA USA; ^3^ Massachusetts General Hospital, Harvard Medical School, Boston, MA USA; ^4^ Center for Alzheimer Research and Treatment, Department of Neurology, Brigham and Women’s Hospital, Boston, MA USA

## Abstract

**Background:**

There is growing interest in preclinical Alzheimer’s disease (AD) trials, as they offer an opportunity to test treatments at early stages of the disease. Yet, there is a lack of data regarding participant feedback on enrollment in these trials. To inform future trial design and retention strategies, we examined participant responses on a Research Satisfaction Survey (RSS) collected as part of the Anti‐Amyloid Treatment in Asymptomatic Alzheimer’s Disease (A4) study, a 4‐year multi‐center preclinical AD trial with a 90‐day screening period (at least 4 visits) and monthly infusion visits.

**Methods:**

The RSS is a self‐reported questionnaire administered at 5 time points that evaluated participant satisfaction with the trial, including aspects that the participants liked most and least. We evaluated the RSS responses at baseline and overtime. For items with sufficient variability in responses, we used generalized linear mixed models with site‐specific random effects to examine potential differences between trial completers and non‐completers in baseline RSS adjusting for age, STAI, family history of dementia, and APOE4 status. As an exploratory approach, we examined trial elements that participants liked most and least based on their ‘negative’ or ‘positive’ responses to repeating their decision to participate in the trial.

**Results:**

In A4,1169 participants were randomized, of which >90% responded positively to all questions on satisfaction throughout the trial. Participants most frequently affirmed the altruism construct, and least frequently affirmed the study design construct at all time points (Table 1). Those who responded negatively at baseline to whether they would repeat their decision to participate in the trial had significantly higher odds of study discontinuation [OR:1.99; CI:1.24, 3.19; p=0.005]. We found no significant associations between responses for any other construct and study completion status. Our exploratory outcomes are summarized in Table 2.

**Conclusion:**

The overwhelming majority of randomized participants reported having a positive experience in this long duration trial that included extensive testing procedures and frequent infusions. Differences in RSS responses may be observed between trial completers and non‐completers at baseline after the lengthy screening process and may guide future retention strategies. Future work will include modeling longitudinal changes throughout the trial.